# Evaluation of Pulmonary Vein Fibrosis Following Cryoballoon Ablation of Atrial Fibrillation: A Semi-Automatic MRI Analysis

**DOI:** 10.3390/jcdd10090396

**Published:** 2023-09-14

**Authors:** Andrea Ballatore, Erika Negrello, Marco Gatti, Mario Matta, Paolo Desalvo, Lorenzo Marcialis, Stefania Marconi, Davide Tore, Massimo Magnano, Arianna Bissolino, Giulia De Lio, Gaetano Maria De Ferrari, Michele Conti, Riccardo Faletti, Matteo Anselmino

**Affiliations:** 1 Division of Cardiology, Cardiovascular and Thoracic Department, Città della Salute e della Scienza Hospital, 10126 Turin, Italy; 2 SC Chirurgia Generale 2, Fondazione IRCCS Policlinico San Matteo, 27100 Pavia, Italy; 3 Radiology Unit, Department of Diagnostic Imaging and Interventional Radiology, Città della Salute e della Scienza Hospital, 10126 Turin, Italy; 4 Dipartimento di Ingegneria Civile e Architettura, Università di Pavia, 27100 Pavia, Italy; 5 Fondazione IRCCS Policlinico San Matteo, 27100 Pavia, Italy

**Keywords:** atrial fibrillation, cryoballoon ablation, cardiac magnetic resonance, fibrosis, recurrence

## Abstract

Current guidelines recommend the use of cardiac magnetic resonance imaging (MRI) for the management of atrial fibrillation (AF). However, the widespread use of cardiac MRI in clinical practice is difficult to achieve. The aim of the present study is to assess whether cardiac MRI can be adopted to identify ablation-induced fibrosis, and its relationship with AF recurrences. Fifty patients undergoing AF cryoballoon ablation were prospectively enrolled. Cardiac MRI was performed before and 30 days after the index ablation. Commercially available software and a specifically designed image processing workflow were used to quantify left atrium (LA) fibroses. Thirty-six patients were finally included in the analysis; twenty-eight were analyzed with the dedicated workflow. Acute electrical isolation was achieved in 98% of the treated pulmonary veins (PVs). After a median follow-up of 16 months, AF recurrences occurred in 12 patients (33%). In both analyses, no differences were found between the subgroups of patients with and without recurrence in the variation of either LA fibrosis or fibrosis at the ostium of the PV, before and after ablation. The ability to predict arrhythmic recurrences evaluated via the ROC curve of the variations in both LA fibrosis (AUC 0.566) and PV fibrosis (AUC 0.600) was low. Cardiac MRI holds the potential to provide clinically significant information on LA disease and AF progression; however, LA fibrosis cannot be easily identified, either by currently available commercial programs or custom tools.

## 1. Introduction

Catheter ablation has proven to be an effective and safe strategy for the management of atrial fibrillation (AF), and has therefore earned stronger recommendations in recent guidelines [1]. Following the evidence that catheter ablation as first-line therapy appears superior to antiarrhythmic drugs (AADs) for rhythm control in patients with symptomatic atrial fibrillation [2], the use of cryoablation [3,4,5,6,7,8] has rapidly increased over the last years. Based on a single-shot approach, cryoballoon ablation allows for the creation of a circular and transmural lesion at the ostium of each pulmonary vein (PV), leading to an easily reproducible electrical isolation (PVI). During the cryoenergy application, the tissue is usually frozen to −40 °C, provoking cell injury and complete isolation between the PV and LA [9]; the achievement of circumferential and transmural fibrosis is at the basis of long-term complete PVI and clinical procedural success.

The role of cardiac magnetic resonance imaging (MRI) has grown extensively in recent years, being crucial for diagnosing different cardiomyopathies, and for risk stratification in patients at risk of sudden cardiac death. In addition, current guidelines also suggest that cardiac MRI should be used almost routinely for a tailored management of patients with AF. Indeed, the newly proposed 4S-AF scheme highlights the need for substrate severity characterization by both clinical and imaging assessment: first by echocardiography, and then by cardiac MRI [1].

The aim of the present study was to assess whether cardiac MRI can be adopted to identify ablation-induced fibrosis at the PV ostium, and whether this relates to AF recurrences.

## 2. Materials and Methods

### 2.1. Study Population 

Consecutive patients undergoing cryoballoon ablation for atrial fibrillation at our center were prospectively enrolled in the study. The main inclusion criteria were the following: age > 18 years old; written informed consent; absence of contraindications to cardiac MRI (e.g., non-MRI compatible device; severe claustrophobia) and to gadolinium administration (e.g., previous allergic reactions; eGFR < 30 mL/min/1.73 m^2^). Arrhythmia recurrences were defined as ECG-documented recurrences at 12-lead ECG and 24-h Holter recordings, or as clinically recognized symptomatic episodes. All of the patients underwent 24-h Holter ECG and outpatient visits at 3 and 12 months, and subsequently they were followed up with telephone interviews. The study was approved by the local ethical committee and conducted according to the Declaration of Helsinki.

### 2.2. Cryoballoon Ablation and Cardiac MRI Acquisition Protocols

Before the ablation procedure, all of the patients underwent transesophageal echocardiography to rule out left atrial appendage (LAA) or LA thrombosis; they also underwent contrast-enhanced cardiac MRI to assess their LA morphology. The cryoballoon ablation protocol adopted at our center has been detailed elsewhere, and is reported in the Supplementary Material. Before and at 30 days after cryoballoon ablation, cardiac MRI was performed with a 1.5-Tesla scanner (Achieva, version 2.6, Philips Medical Systems, Eindhoven, The Netherlands) using a 32-channel body phased-array coil. Paramagnetic contrast medium (Prohance, gadoteridol, Bracco Imaging, Milan, Italy) was administered at a dose of 0.1 mmol/kg. The following sequences were acquired:

- Angio-MRI (non ECG-gated, 4D time-resolved with keyhole 4D-TRAK);
- LGE-MRI (free-breathing navigator and ECG-gated inversion recovery gradient-echo sequence);

Detailed descriptions of the parameters used for each sequence are reported in the Supplementary Material.

### 2.3. Fibrosis Quantification

**Figure 1:**
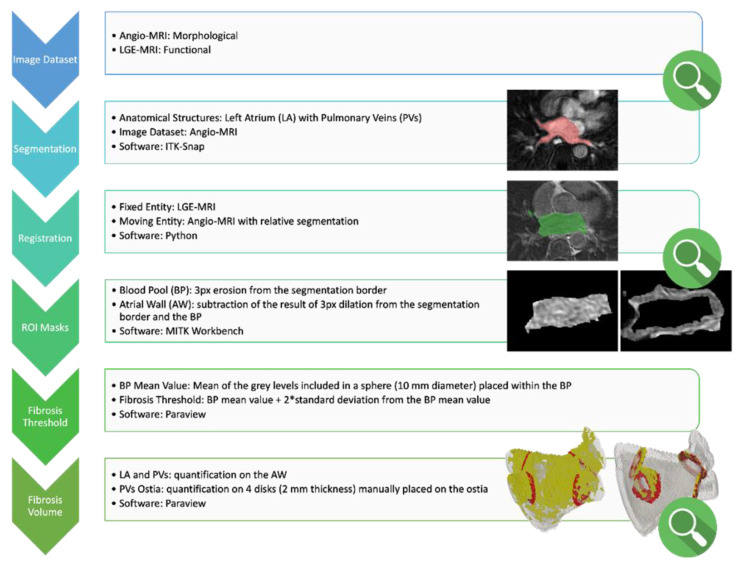
Custom study specific workflow for image processing and fibrosis quantification. Red and green area in the “Segmentation” and “Registration” steps, respectively, refer to the region of interest analyzed in the corresponding step. Please refer to “Figure 4” legend for the images in the “Fibrosis Volume” phase. Please refer to the main text for the version number of the software indicated in the figure. “*”: multiplication sign.

ADAS 3D LA (Galgo Medical, Barcelona, Spain) was used for the quantification of LA fibrosis at baseline and after the ablation, as previously described [10], applying validated thresholds to discriminate normal from fibrotic atrial tissue [11]. Only the data on core fibrosis were collected and analyzed. Subsequently, we developed a dedicated image processing workflow (Figure 1) to ensure volumetric quantification of the amount of ablation-induced fibrosis.

Below are the details of each step of the established workflow.

1. Image selection: For each patient, the available dataset is composed of the following:
  - Angio-MRI images, which mainly provide anatomical information. This sequence has been used for 3D reconstruction.
  - LGE-MRI sequences that provide the best functional information about fibrosis, thanks to the use of a paramagnetic contrast agent. This sequence has been used for fibrosis quantification.
2. Image segmentation: Left Atrium (LA) and Pulmonary Veins (PVs) segmentation are semi-automatically obtained from angio-MRI images by means of ITK-Snap (v. 3.8) software. Segmentation is a crucial step, since the success of the whole analysis depends on its quality. In some cases, a manual correction of the segmentation is required. The operator who performed the segmentations is an expert in medical image processing with anatomical knowledge in the region of interest.
3. Image registration: An in-house Python routine is implemented to perform image registrations: patient’s LGE-MRI is used as the fixed image, while angio-MRI and its segmentation are the moving ones. The transformations allows alignment of multiple images acquired from different modalities, and combines them into the same coordinate space. Thus, it is possible to merge together anatomical and functional information to recognize each tissue’s characteristic in a precise locus. The output of the registration step is the LA and PVs segmentation properly located on LGE-MRI. The atrium identification on the functional MRI allows one to start with the fibrosis analysis.
4. Blood pool and LA wall identification: Different regions of interest (blood pool and LA wall) are isolated using MITK Workbench (v. 2022.04) software. Starting from the registered atrium segmentation, the area identifying the blood pool is delineated by eroding the segmentation contour of 3 pixels, employing sphere morphology. Following the same method, the atrial wall is extracted. By performing a Boolean subtraction between the dilated and the eroded contour (3 pixels with sphere morphology), an area that likely includes the atrial wall is determined. Masking the LGE-MRI image with both the previously identified regions (blood pool and LA wall), an image with only the pixels included in that area is obtained. This kind of image—exported in .vtp format—is feasible to be analyzed with an in-house-developed ParaView (v. 5.11.0) routine.
5. Fibrosis threshold selection: The threshold to discriminate between normal tissue and fibrotic tissue is set on each image as the average gray level value of a 10 mm diameter sphere placed within the blood pool + 2 standard deviation. The choice to use a sphere of a fixed diameter to isolate the pixels with the blood pool is made because of standardization, and to ensure the selection is always in a blood pool region, regardless of the accuracy of the segmentation.
6. Fibrosis volume measurement:
  - LA and PVs wall: Applying the threshold on the gray levels included in the fictional atrial wall, the amount (volume) of fibrosis on the whole left atrium together with PVs is measured.
  - PVs ostia: To isolate the fibrosis volume on each PV (right and left upper pulmonary veins and right and left lower pulmonary veins), the user has to position four disks of 2 mm thickness in correspondence to the four PVs ostia. By isolating the four regions, the amount of fibrosis on each PV ostium can be measured.
7. Qualitative evaluation steps: The evaluation steps during the workflow are performed manually by a radiologist. Three evaluation steps are required: the first one is to check the quality of the available image datasets (angio-MRI and LGE-MRI). A second evaluation is needed after registration of the angio-MRI sequence and atrium segmentation on LGE-MRI. The last evaluation step is required to verify the correct position of the marker for the isolation of the PV ostia, with the aim of measuring the localized fibrosis.

The following parameters were measured at baseline and at 30 days after the index procedure:

- LA total fibrosis indexed on LA surface and/or volume;
- Fibrosis at the ostium of each PV;
- Surface and/or volume of atrial tissue at the ostium of each PV.

An increase in LA fibrosis was defined as the difference between total fibrosis before and after the ablation normalized for baseline fibrosis. An increase in PV ostial fibrosis was defined as a difference in fibrosis measured at the PV level indexed on the PV surface and/or volume. The number of PVs without an increase in ostial fibrosis was defined as the number of PVs per patient with a negative difference in ostial fibrosis after the ablation compared to baseline.

### 2.4. Statistical Analysis

The categorical variables are reported as percentages. The continuous variables are reported as mean ± standard deviation (SD), or median and interquartile range (IQR). Student’s *t*-test and Fisher’s exact test were used to compare the continuous and categorical variables between groups, respectively. Logistic regression and receiver operating characteristic (ROC) curves were used to analyze the relationship between LA and PV ostial fibrosis, and the number of PVs without an increase in fibrosis after the ablation and arrhythmic recurrences. Survival analyses were performed with the Kaplan–Meier curve and log-rank tests. A two-tailed *p*-value < 0.05 was considered statistically significant. All analyses were performed using R software version 4.2.2 (R Foundation for Statistical Computing, Vienna, Austria).

## 3. Results

Starting October 2018, 50 patients were prospectively enrolled in the study. Cardiac MRI at 30 days was not performed in six patients, and in three cases due to logistics related to the COVID-19 pandemic. There were 11 (22%) and 16 (32%) patients who were not analyzed with the ADAS and the custom workflow analyses, respectively, due to poor MRI quality and/or an inability to process the images to quantify fibroses reliably.

The baseline characteristics of the 36 patients finally included in the analysis are reported in Table 1. The mean age was 60.5 years; 39% of the patients were female, and the majority suffered from paroxysmal AF (92%); the mean number of previous AADs or rate control agents was 1.4 different agents per patient.

At baseline, the mean indexed LA volume was 41 mL/mq. According to Utah classification (ADAS analysis), 45% of the patients were classified as stage 1, 36% as stage 2, 3% as stage 3, and 27% as stage 4; overall, the mean LA fibrosis was 14.2%.

Acute electrical isolation was achieved in 98% of the PVs. After a median follow-up of 16 months, AF recurrences occurred in 12 patients (33%). Symptomatic episodes were reported in two patients: one patient declared self-terminating episodes lasting around 24 h, similar to those prior to the ablation; the other patient suffered a symptomatic episode similar to those prior to the ablation, lasting several hours, which terminated on assumption of oral flecainide. All of the remaining patients were categorized as AF recurrences due to arrhythmia documentation at 12-lead ECG or 24-h Holter recordings during follow-up. No statistically significant differences in baseline characteristics were found between patients with and without recurrences, with the exception of a greater use of beta-blockers both at baseline (83% vs. 17%, *p*-value < 0.001) than during follow-up (65% vs. 25%, *p*-value = 0.035) in patients who were free from arrhythmic recurrences (Table 1).

### 3.1. Fibrosis Quantification Using ADAS 3D LA Software

No difference in baseline LA fibrosis was found between patients with and without AF recurrence (12.0 vs. 15.3%, *p* = 0.332). Neither LA or PV ostial fibrosis significantly increased after the index procedure (Table 2). Similarly, freedom from AF recurrences was not associated with a difference in LA, PV ostial, or number of PVs without an increase in fibrosis after the ablation, with both the univariate and multivariate analyses (Table 3).

### 3.2. Fibrosis Quantification Using a Novel Dedicated Image Processing Workflow

Overall baseline LA fibroses appeared to be lower in patients with AF recurrences compared to those free from arrhythmia (4.1 vs. 8.1%, *p* = 0.044). A nonsignificant difference was found between the subgroups of patients with and without recurrence in the variation of either LA or PV ostial fibroses before and after ablation (Table 2). Neither the univariate or multivariate analysis baseline for LA fibrosis was related to AF recurrences for the difference before and after the procedure of LA and PV ostial fibrosis, nor the number of PV without an increase in fibrosis after the ablation (Table 3).

**Figure 2:**
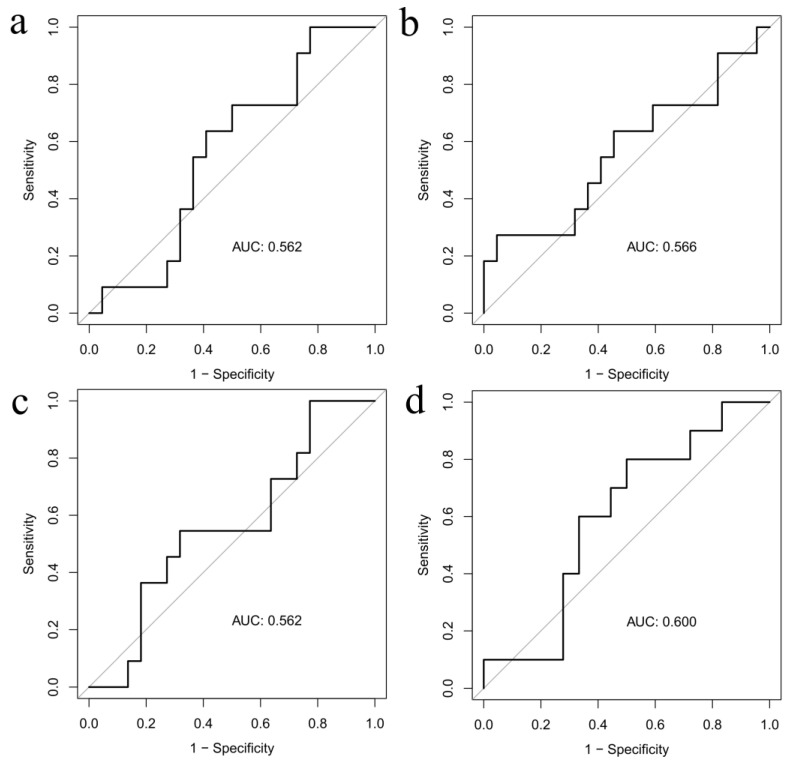
ROC curve for increase in LA (panels **a** and **b**) and PV ostial (panels **c** and **d**) fibroses by ADAS and custom workflow, respectively.

The ability to predict arrhythmic recurrences evaluated by the ROC curves of the variation before and after the index procedure of both LA fibroses (AUC 0.566 and AUC 0.562) and ostial PV fibroses (AUC 0.600 and AUC 0.562) was low (Figure 2), for both the custom and the ADAS workflows.

**Figure 3:**
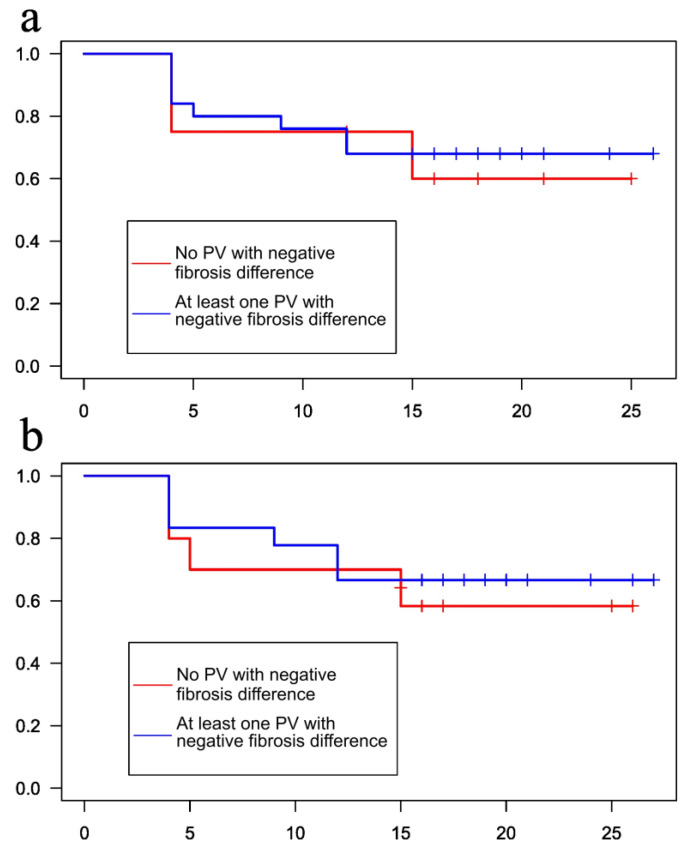
Kaplan–Meier arrhythmia-free survival curves stratified according to the presence of at least one PV with (red line) or without (blue line) increase in ostial PV fibrosis after the procedure by ADAS (panel **a**) and custom (panel **b**) workflows, respectively.

Finally, the absence of one or more PVs without an increase in ostial fibrosis after the ablation did not correlate with improved freedom from arrhythmia recurrences at follow-up (60% vs. 68%, *p*-value = 0.8 for ADAS analysis, and 58.3% vs. 66.7%, *p*-value = 0.7 for the custom image processing workflow, Figure 3).

## 4. Discussion

The main findings of the present analysis are the following:

- Evaluation of atrial fibrosis using MRI sequences commonly used in clinical practice is challenging and not routinely feasible;
- Assessment of PVI beyond the acute phase via tissue characterization with MRI does not seem viable at present.

Current guidelines recommend the use of imaging techniques to identify LA remodeling associated with AF1; in particular, MRI is indicated within the methods to assess LA anatomy, tissue structure, function, as well as detect LA or LAA thromboses. In addition, cardiac MRI provides information on the anatomical relationships between the esophagus and the heart, which are particularly useful in planning the ablation procedure [12,13,14,15].

Several elements have been identified as risk factors for AF recurrence after catheter ablation, including LA diameter, early recurrences, and valvular AF [16]. On top of these classical risk factors, the DECAAF study [17] showed that the burden of atrial fibrosis detected with pre-operative MRI correlated with increased risk of arrhythmia recurrences. On these bases, the DECAAF II trial [15] investigated the role of MRI-guided fibrosis ablation in addition to PVI in patients with persistent AF; however, the more extensive ablation strategy was not associated with improved arrhythmia control, and additionally carries a higher risk of ischemic stroke. Several differences exist between the present research and the DECAAF study, in which baseline fibrosis was assessed and related to the occurrence of AF recurrences. As reported in the Results section, even in the present analysis, some grade of baseline fibrosis was detected among patients with and without recurrences. Nevertheless, the aim and protocol of DECAAF and our study are profoundly different. Aim of the present study was to quantify ablation-induced atrial fibrosis at a specific anatomical site. Thus, the inability to accurately quantify fibroses at a site in which cryoablation has definitely been applied suggests a technical challenge in identifying LA fibrosis at MRI. Moreover, the negative results of the DECAAF 2 trial support the hypothesis that fibroses cannot be easily identified and used to guide the ablation strategy.

Overall, the routine use of fibrosis quantification by means of LGE-MRI presents several difficulties; even in the best-case scenario of a clinical study, in almost 20% of patients, poor MRI quality did not allow for fibrosis quantification [17,18]. Moreover, even if theoretically good correlations are presented, different methods for quantifying fibroses can yield different results for the fibrosis burden [18].

Previous MRI studies have analyzed the extension of fibrosis induced by PVI, but they focused especially on RF ablations. A complete circumferential fibrosis in all of the PVs was found in a minority of treated patients, and a clear relationship between circumferential fibrosis and long-term procedure success did not always emerge [19,20,21,22]. Similar results were obtained in studies on cryoballoon ablation [23,24,25,26]. Moreover, dense scarring and interstitial fibrosis can be difficult to differentiate via MRI, potentially leading to inaccurate estimations of lesion gaps at the PV ostium. A recent analysis on 19 patients undergoing cryoballoon ablation found that the absence of a major gap in any PV was associated with freedom from AF recurrence at 12 months [26]. However, in this experience, a qualitative rather than a quantitative definition of circumferential lesion was adopted, allowing for a gap of up to one-third of the PV ostium for the definition of complete PV fibrosis; moreover, a significant percentage of patients with major gaps were nonetheless free from arrhythmia recurrences. A possible explanation for these contradictory results is that cryoballoon ablation may have effects on extracardiac structures (e.g., parasympathetic ganglia) that cannot be assessed via cardiac MRI and nevertheless result in additional antiarrhythmic effects.

ADAS 3D software (Galgo Medical, Barcelona, Spain) is currently the state-of-the art among commercially available programs, for evaluation of both atrial and ventricular cardiac fibroses. Its role in fibrosis quantification and planning of ventricular tachycardia ablation is well-established. Nevertheless, atrial fibrosis quantification entails greater challenges, as the atrial wall is thinner than the ventricular. Moreover, the fibrotic remodeling is diffused without evidence of a clear and defined scar, as is found in ischemic cardiomyopathy. Indeed, in our cohort, we failed to identify a significant increase in fibrosis induced by cryoballoon ablation irrespective of arrhythmia recurrence status, using the ADAS 3D LA software.

**Figure 4:**
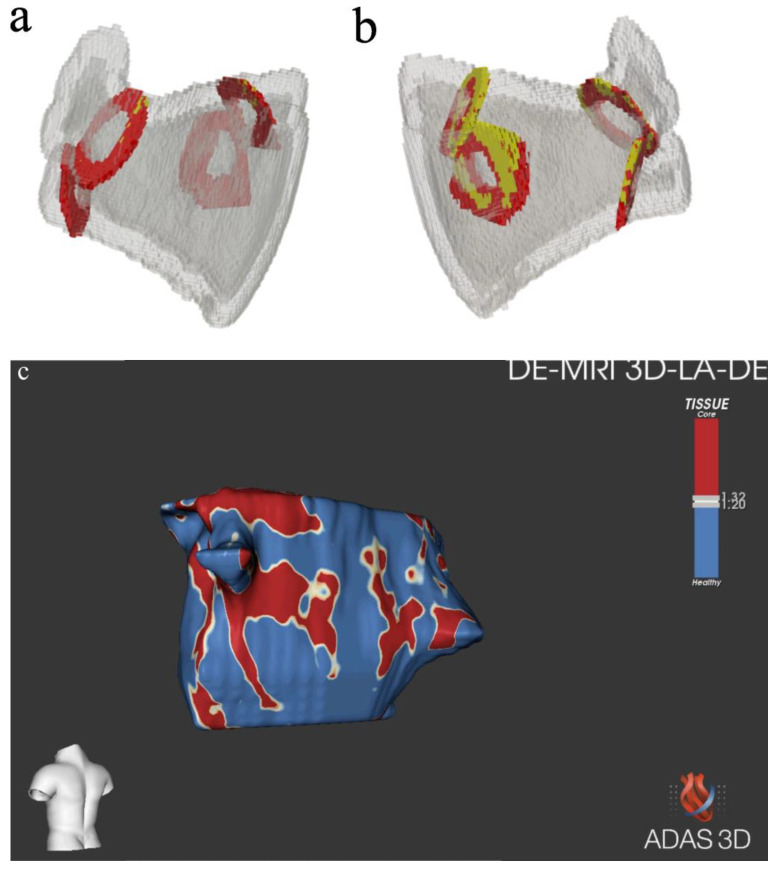
Pre-ablation (panel **a**) and post-ablation (panel **b**) reconstruction of the LA using the custom study’s specifically developed workflow. Region of interest representing the PV ostia are represented in red; PV ostial fibrosis is represented in yellow. Reconstruction of atrial anatomy and fibrosis (panel **c**) with ADAS software [images were obtained with the ADAS 3DTM (ADAS3D Medical, Barcelona, Spain)].

For this reason, we developed a workflow specifically tailored to assess LA fibrosis based on the acquired MRI sequences (Figure 4, Panels a and b). This approach had the potential to optimize fibrosis identification in a semi-automatic manner, combined with the direct observations of experienced radiologists or cardiologists. Nonetheless, similarly to the ADAS analysis (Figure 4, Panel c), no significant differences emerged after the index procedure in fibrosis at the PV ostia was performed. The differences in detected values (Table 2) between our custom workflow and ADAS analysis may be attributable to the fact that our workflow analyzed the fibrosis as a volume, whereas ADAS software did so as a surface.

### Limitations

This research presents the following limitations. The small cohort limits the possibility of generalizing the results to a broader population, and may be statistically underpowered, preventing detection of significant differences. Possible extracardiac effects of cryoablation could not be assessed with the present analysis. Similarly, the data heterogeneity and dispersion were not marginal (Supplementary Figures S1 and S2) and may, at least partially, explain some of the observations described. Since radiofrequency ablations were not performed in these patients, considerations on how the analyzed software could have performed in that scenario cannot be derived. Moreover, the detection threshold of the MRI equipment must be taken into account. Finally, a non-marginal quote of MRI could not be analyzed; nevertheless, this finding is in line with previous reports, and highlights the difficulties of routinely implementing LGE analysis for fibrosis quantification and AF management in daily clinical practice.

## 5. Conclusions

Cardiac MRI holds the potential to provide several clinically significant details on LA disease and AF progression. However, there are currently several technical constraints that prevent the widespread adoption of cardiac MRI for AF characterization in clinical practice, since LA fibrosis cannot be easily identified. Therefore, further studies are needed before allowing the routine adoption of cardiac MRI in this setting, a step that would surely represent a critical advancement towards successful AF management.

## Figures and Tables

**Table 1:** Baseline characteristics of the included patients stratified by arrhythmia recurrence. The reported *p*-values refer to comparisons between recurrence subgroups.

Variable	General Population	Arrhythmic Recurrence	No Recurrence	*p*-Value
Age	60.5 (±9.7)	58.3	61.7	ns
Gender (female)	14 (38.9%)	5 (41.7%)	9 (37.5%)	ns
Paroxysmal AF	33 (91.7%)	12 (100%)	20 (83.3%)	ns
AF history duration (months)	78.3 (±85.2)	114.6	61.9	ns
AF at the procedure	9 (25.0%)	4 (33.3%)	4 (16.7%)	ns
Hypertension	16 (44.4%)	5 (41.7%)	11 (45.8%)	ns
Diabetes	3 (8.3%)	2 (16.7%)	1 (4.2%)	ns
Previous stroke	1 (2.7%)	1 (8.3%)	0	ns
CAD	1 (2.7%)	0	1 (4.2%)	ns
Thyroid disorders	3 (8.3%)	1 (8.3%)	2 (8.3%)	ns
*Prior use of AADs*				
Amiodarone	2 (5.6%)	1 (8.3%)	1 (4.2%)	ns
Flecainide	8 (22.2%)	1 (8.3%)	7 (29.2%)	ns
Propafenone	12 (33.3%)	5 (41.7%)	7 (29.2%)	ns
Sotalol	4 (11.1%)	3 (25%)	1 (4.2%)	ns
Beta-blockers	23 (63.9%)	2 (16.7%)	20 (83.3%)	<0.001
Digoxin	0 (0%)			ns
*Oral anticoagulants*				
VKA	2 (5.6%)	0	2.3 (8%)	ns
DOAC	23 (63.9%)	7 (58.3%)	16 (66.7%)	ns
Indexed LA volume mL/mq	41.1 (±10.2)	40.3	41.5	ns

AAD: anti-arrhythmic drugs; AF: atrial fibrillation; CAD: coronary artery disease; DOAC: direct oral anticoagulation; LA: left atrium; ns: non-significant; VKA: vitamin K antagonist.

**Table 2:** Comparison of LA fibrosis and fibrosis at the PV ostium before and after ablation.

Variable	Subgroup		*p*-Value
	Recurrence	No Recurrence	
Analysis based on ADAS			
Increase in LA fibrosis after ablation	1.7	1.9	0.885
Increase in PV fibrosis after ablation	0.10	−0.10	0.415
Number of PVs without an increase of fibrosis after ablation	1.7	2.1	0.509
Analysis based on the custom study specific workflow			
Increase in LA fibrosis after ablation	10.8	1.8	0.243
Increase in PV fibrosis after ablation	0.30	0.15	0.422
Number of PVs without an increase of fibrosis after ablation	1.1	1.6	0.393

LA: left atrium; PV: pulmonary vein. Increase in LA fibrosis after ablation is reported as the difference between LA fibrosis at the follow-up MRI and baseline LA fibrosis, indexed on baseline LA fibrosis. Increase in PV fibrosis is reported as the difference between PV fibrosis at the follow-up MRI and baseline PV fibrosis, indexed on baseline PV fibrosis.

**Table 3:** Odds ratio of univariate and multivariate analyses predicting AF recurrences.

Variable	Univariate	Multivariate
Analysis based on ADAS		
Baseline LA fibrosis	0.99 (95%CI: 0.93–1.04)	0.99 (95%CI: 0.91–1.08)
Increase in LA fibrosis after ablation	0.99 (95%CI: 0.80–1.17)	0.90 (95%CI: 0.65–1.16)
Increase in PV fibrosis after ablation	1.48 (95%CI: 0.54–4.56)	1.16 (95%CI: 0.14–10.79)
Number of PVs without an increase of fibrosis after ablation	0.83 (95%CI: 0.49–1.40)	0.75 (95%CI: 0.23–2.23)
Analysis based on the custom study specific workflow		
Baseline LA fibrosis	1.00 (95%CI: 1.00;1.00)	1.00 (95%CI: 1.00;1.00)
Increase in LA fibrosis after ablation	1.08 (95%CI 1.00–1.28)	1.05 (95%CI 0.98–1.29)
Increase in PV fibrosis after ablation	1.01 (95%CI 0.99–1.03)	1.00 (95%CI 0.97–1.03)
Number of PVs without an increase of fibrosis after ablation	0.76 (95%CI 0.39–1.38)	1.10 (95%CI 0.39–3.00)

CI: confidence interval; LA: left atrium; PV: pulmonary vein.

## Data Availability

The data underlying this article will be shared on reasonable request to the corresponding author.

## Supplementary Materials

The following supporting information can be downloaded at: https://www.mdpi.com/article/10.3390/jcdd10090396/s1, Figure S1: Increase in LA (panel A) and PV (panel B) fibrosis after the ablation according to our dedicated workflow analysis; Figure S2: Increase in LA (panel A) and PV (panel B) fibrosis after the ablation according to ADAS analysis; Section S1: Procedural details; Section S2: Cardiac MRI sequences acquisition parameters.
