# Supplementary material for: Evaluation of Pulmonary Vein Fibrosis Following Cryoballoon Ablation of Atrial Fibrillation: A Semi-Automatic MRI Analysis
Source: J Cardiovasc Dev Dis. 2023 Sep 14;10(9):396. doi: 10.3390/jcdd10090396 (PMC10531798; doi:10.3390/jcdd10090396)

Evaluation of pulmonary vein fibrosis following cryoballoon ablation of atrial fibrillation: a semi-automatic MRI analysis

Supplementary Material

Procedural details

Transesophageal echocardiography and a pre-procedure contrast enhanced cardiac MRI were performed in all patients to rule out atrial thrombi and study the LA morphology, respectively. All procedures were executed in conscious sedation using boluses of midazolam and fentanyl, and exclusively under fluoroscopy guidance. Surface ECG and intracardiac electrograms were recorded using EP-WorkMate (St. Jude Medical, Saint Paul, USA). Through venous femoral accesses the catheters were introduced to the heart. A diagnostic decapolar electrophysiological catheter was placed in the coronary sinus (CS), subsequently access to the LA was achieved through a patent foramen ovale or by single transseptal puncture. Heparin was administered intravenously at the moment of the transseptal puncture, and during the procedure to achieve the target activated clotting time (ACT > 350 s). A steerable 15Fr sheath (FlexCath Advance, Medtronic) was used to introduce a 28-mm cryoballoon ablation catheter (Arctic Front Advance, Medtronic) and an inner lumen mapping catheter (Achieve, Medtronic) in the LA and subsequently in each PV ostium. Complete occlusion was verified by selective venography, when no medium contrast leak back in the LA was observed. According to our standard approach, cryoenergy was delivered at each PV ostium for 180 seconds if PV isolation was achieved in less than 60 seconds, for 240 seconds for the other cases. However, the electrophysiologist was free to adopt different ablation scheme based on the temperature curve profile or other factors. Whenever the minimum temperature reached -55°C the ablation was stopped. In order to avoid phrenic nerve palsy, during ablation of the right PVs the catheter in the CS was positioned in the right innominate vein or subclavian vein and the ipsilateral phrenic nerve paced at 800-1200 ms cycle. Cryoenergy application was immediately stopped if a loss of diaphragmatic stimulation was detected. The patients were discharged from hospital the day after ablation if the clinical conditions were stable.

Cardiac MRI sequences acquisition parameters

1. Angio-MRI (non ECG-gated, 4D time resolved with keyhole 4D-TRAK)

Parameter	Standard value
TR	4,035 ms
TE	1,27 ms
Flip Angle	30°
Acquisition matrix	220*220
Acquisition voxel	1,50/ 1,50/ 3,00 mm
Recon voxel	0,76/ 0,76/ 1,50 mm
FOV AP	330 mm
FOV RL	330 mm
FOV FH	112 mm

2. LGE-MRI (free breathing navigator and ECG gated inversion recovery gradient-echo sequence)

Look Locker sequences were used prior to LGE-MRI acquisition to identify the best inversion time for LGE imaging.

3D Inversion Recovery	Standard value
TR	4,25 ms
TE	1,33 ms
Flip angle	15°
IT	340 ms
Acquisition matrix	200*252
Acquisition voxel	1,39/ 1,59/ 2,80 mm
Recon voxel	0,89/ 0,89/ 1,40 mm

Supplementary Figure S1. Increase in LA (panel A) and PV (panel B) fibrosis after the ablation according to our dedicated workflow analysis.

A)

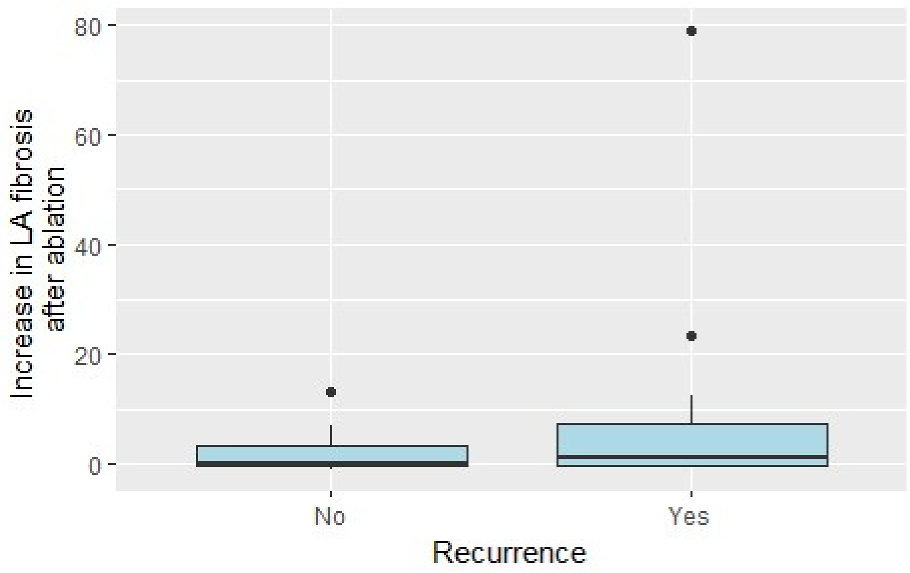

B)

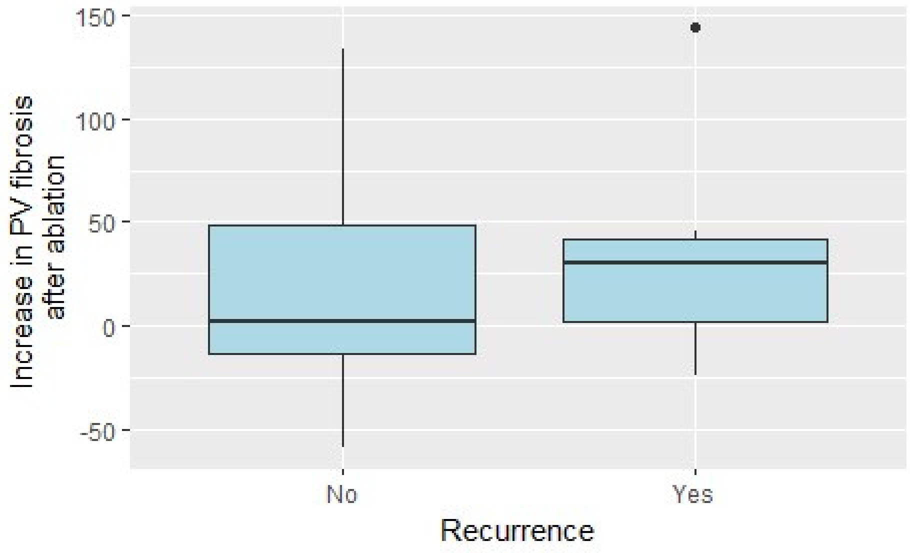

x

Supplementary Figure S2. Increase in LA (panel A) and PV (panel B) fibrosis after the ablation according to ADAS analysis.

A)

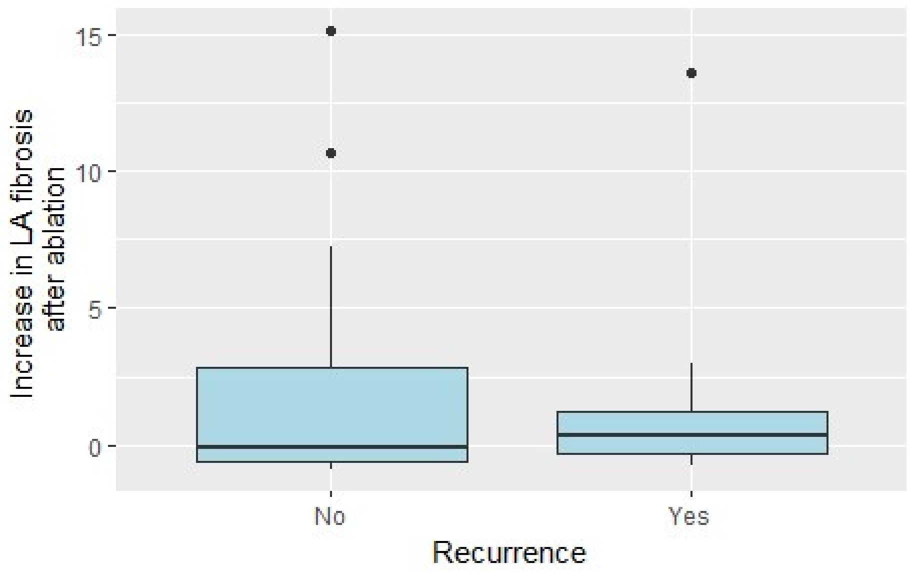

B)

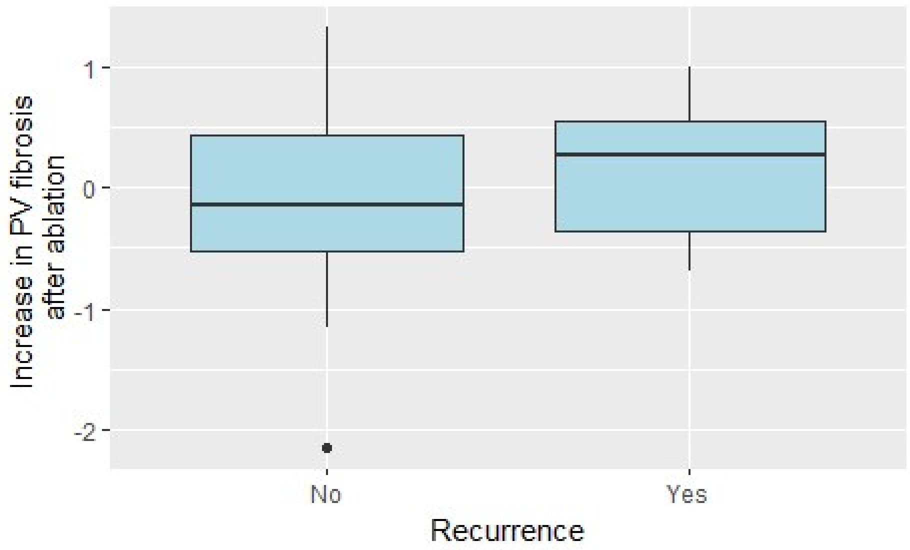

Supplement: Supplementary file 1 [file jcdd-10-00396-s001.zip › jcdd-2506585-supplementary.pdf]
